# Seeing is believing

**DOI:** 10.1038/s41377-022-00737-4

**Published:** 2022-02-28

**Authors:** Sergey Krasikov, Yuri Kivshar

**Affiliations:** Nonlinear Physics Center, Research School of Physics, Australian National University, Canberra ACT 2601, Australia and School of Physics and Engineering, ITMO University, St. Petersburg, 197101 Russia

**Keywords:** Metamaterials, Nanophotonics and plasmonics

## Abstract

Bound states in the continuum are realized in many optical systems as “dark states”, and their presence can be detected in the regime of leaky modes via resonances in far-fields. Here the authors reveal previously unseen structure of bound states in the continuum by exploring strong near-field localization in dielectric metasurfaces.

Bound states in the continuum (BICs) originate from earlier days, when two pioneers of quantum mechanics^1^ discovered that certain potentials in the Schrödinger equation describing a quantum particle can support spatially localized (bounded) states with the energies larger than the maximum energy of the potential, i.e., their energy belong to the radiation spectrum. For many years, such exotic radiationless states were considered as a mathematical curiosity, however later it was discovered that such states can occur in many different physical systems that support waves interacting predominantly outside a potential due to destructive interference effects. Because of the generality of wave phenomena and interference, BICs can be observed for different types of waves, including water waves and acoustic waves, and they have been shown to exist in many dissimilar optical systems ranging from waveguides to photonic crystals^2^.

In optics, BICs are usually observed as “quasi-BICs” when exact parameter matching is not satisfied (e.g., due to a finite size of a system), and destructive interference of leaky waves can be detected in far-fields as a sharp resonance with a high value of the quality factor (Q factor) (see, e.g., the earlier review paper^3^). Such ultra-narrow resonances can be utilized for many applications, for example, for biosensing^4^, lasing^5^, and nonlinear photonics^6^. The key idea behind the common physics of different and dissimilar BICs is a suppression of radiative losses via destructive interference of leaky modes and, since this suppression can never be made complete, BICs in physical systems are always manifested as quasi-BICs because otherwise *they are not seen in far-fields*. Fig. 1

**Fig. 1:**
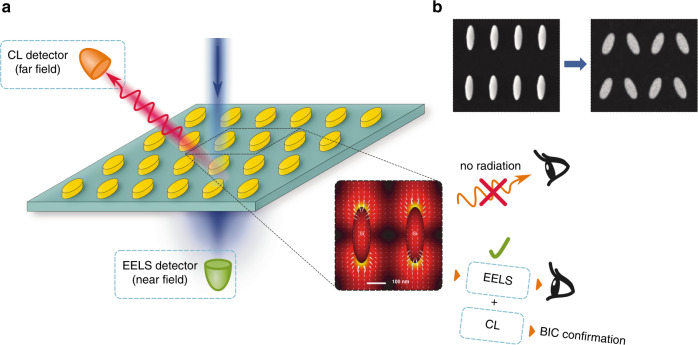
Schematic for exciting and probing bound-states-in-the-continuum (BIC) modes. **a** Experimental STEM setup is used to focus a high-energy electron beam to probe a dielectric metasurface. Complementary measurements of the energy lost by electrons, and energy of emitted photons result in EELS and CL spectra respectively. **b** Transformation of BIC into quasi-BIC

The dark nature of a BIC state is simultaneously one of its main features and largest weakness. Together with high values of the Q factor, the absence of coupling with far-fields does not allow to access such an optical state for a direct characterization. Instead, all practical applications utilizing BICs rely on “an image” of quasi-BIC in the far-field region.

A new milestone in the study of BICs comes with the recent work by Dong et al.^7^ who suggested a way *to observe true BICs via direct probing techniques* utilizing a combination of two types of spectroscopies. More specifically, they considered a metasurface consisting of a lattice of tilted and straight pairs of silicon nanoantennas that support BIC resonances, as suggested in Ref. ^8^.

Dong et al^7^. realized experimentally a combination of *cathodoluminescence* (CL) and monochromatic *electron energy loss spectroscopy* (EELS) in a scanning transmission electron microscope (STEM). They employed a focused electron beam to excite specific optical modes in the near-field region. Importantly, the energy transferred to both radiative and nonradiative modes can be measured with EELS, while only radiative losses are measured with CL in the far-field region. A comparison of both EELS and CL spectra allows “to see” directly both leaky and trapped optical modes, thus characterizing directly *true photonic BIC states* with a nanometer accuracy. More specifically, if a high-Q resonance is observed in the EELS spectra but, at the same time, it does not occur in the far-field region, this provides direct evidence of a true BIC-induced “darkness of light”.

Additional important capability enabled by the developed tool is the possibility to determine directly coherent interaction length of a quasi-BIC state and compare it with the collective effects in the antenna array. For that, CL spectra are analyzed as a function of the distance to the edge of the array. At the resonant frequency of the quasi-BIC state, the CL emission decreases with a shift of the electron beam from the centre of the structure toward its edge until any sign of quasi-BIC disappears at the outer antenna of the array. Such an effect was not observed for Mie resonances supported by the same structure.

The proposed technique provides an efficient tool for the rapidly developing concepts of high-Q optical devices. We believe that further advances in fundamental research in optical metasurfaces and technological innovations in flat optics will uncover their tremendous potential making a rapid impact on science, technology, and society. In addition, there are many other areas in physics and photonics which will benefit from high-Q metasurfaces supporting BIC resonances and undergo rapid development. Widely regarded as practically unachievable, true BICs can be revealed now. And no matter how dramatic it sounds, only the time will show what else can be found in the dark.

## References

[1] von Neumann J, Wigner EP (1929). Über merkwürdige diskrete Eigenwerte. Physikalische Z..

[2] Koshelev K, Bogdanov A, Kivshar Y (2020). Engineering with bound states in the continuum. Opt. Photon. N..

[3] Hsu CW (2016). Bound states in the continuum. Nat. Rev. Mater..

[4] Tittl A (2018). Dielectric metasurfaces for surface-enhanced spectroscopies. Opt. Photon. N..

[5] Hwang MS (2021). Novel non-plasmonic nanolasers empowered by topology and interference effects. Nanophotonics.

[6] Pertsch T, Kivshar Y (2020). Nonlinear optics with resonant metasurfaces. MRS Bull..

[7] Dong Z (2022). Nanoscale mapping of optically inaccessible bound-states-in-the-continuum. Light.: Sci. Appl..

[8] Koshelev K (2018). Asymmetric metasurfaces with high-*Q* resonances governed by bound states in the continuum. Phys. Rev. Lett..

